# Recent Advances in Mesothelioma Treatment: Immunotherapy, Advanced Cell Therapy, and Other Innovative Therapeutic Modalities

**DOI:** 10.3390/cancers17040694

**Published:** 2025-02-18

**Authors:** Ratoe Suraya, Tatsuya Nagano, Motoko Tachihara

**Affiliations:** Division of Respiratory Medicine, Department of Internal Medicine, Kobe University Graduate School of Medicine, Kobe 650-0017, Japan; dr.ratoesuraya@yahoo.com (R.S.); mt0318@med.kobe-u.ac.jp (M.T.)

**Keywords:** mesothelioma, immunotherapy, immune checkpoint inhibitors, CAR-T cells, dendritic cells, molecular targeted therapy

## Abstract

Mesothelioma is a highly malignant condition arising from the pleura and peritoneum that is closely related to asbestos exposure. The prognosis for this condition has traditionally been poor due to the difficulty physicians have faced in diagnosing and treating this disease, even in its early phase. Fortunately, recent advances in both the molecular understanding of the development of this disease and innovative and novel treatment modalities have accelerated the discovery of new ways to treat mesothelioma. In this review, we first summarize the mechanism of mesothelioma pathophysiology and then relate it to emerging treatment modalities. These include immunotherapy or immune checkpoint inhibitors (ICIs), molecular targeted therapies, and cell-based therapies (such as CAR-T cells or dendritic cells). The scientific basis for the utilization of these treatment modalities, alongside the current clinical evidence for each option, will be explored in detail later on. The hope is that this review can elucidate how these emerging therapeutic options work clinically to help accelerate further developments in novel mesothelioma treatment modalities.

## Simple Summary

The prognosis for highly cancerous mesothelioma patients is known to be poor due to the rapid progression of the disease and the limitations in the treatment options available to physicians. Recent breakthroughs in treatment strategies have led to the emergence of new therapies that may improve the outcomes of mesothelioma patients. These therapies include targeting immune checkpoint inhibitors or specific molecular targets using drugs or modified immune cells that can act to destroy cancer cells, and many other novel strategies are expected in the future.

## 1. Introduction

Mesothelioma is a malignant condition arising from the pleura and peritoneum that is closely related to asbestos exposure [1]. Due to the prolonged time needed for one to fully develop mesothelioma, difficulty in diagnosing this condition early, its quick progression once manifested, and the limited treatment options, the prognosis for this condition has traditionally been poor [2,3]. This review will briefly reflect on what we know about mesothelioma so far and relate it to the novel therapeutic strategies that have been developed over recent years. These include immunotherapy or immune checkpoint inhibitors (ICIs), molecular targeted therapies, and the use of cell-based therapy (such as CAR-T cells or dendritic cells), among others. Mechanistic rationales and clinical evidence will be provided, and prospective therapies will also be discussed.

## 2. Mesothelioma

While the exact mechanism that causes the carcinogenesis of mesothelioma is unknown, the exposure to and inhalation of asbestos fiber is a definite trigger [3]. Around 85% of mesothelioma cases are attributable to occupational asbestos exposure, although only around 10% of those exposed to asbestos actually develop mesothelioma [1]. Moreover, mesothelioma has a long latency period (20–50 years). Thus, even with stringent regulations and bans on asbestos usage, new cases of mesothelioma are still being diagnosed [4,5]. Chronic and repeating cycles of inflammation and healing in the mesothelium have been linked to the molecular mechanism of mesothelioma. Indeed, prolonged exposure to asbestos causes multiple changes in mesothelial cells, including DNA damage and cell cycle arrest, apoptosis defects, and the release of reactive oxygen species and reactive nitrogen species, among others [6,7,8,9]. Asbestos also induces mast cells to release inflammatory mediators such as tumor necrosis factor α (TNF-α), which together can trigger the activation of the central inflammation response protein in nuclear factor-kB (NF-kB) [10]. All of these processes lead to genotoxicity and may cause mutations in mesothelial cells. Prolonged, repeated exposure to asbestos and the subsequent triggering of the above process are currently thought to be the mechanisms behind mesothelioma [3].

The treatment options for mesothelioma have traditionally been limited. For instance, surgery for pleural mesothelioma is controversial because until recently no phase 3 trials had addressed its efficacy. Selecting patients who may benefit from surgery is a key component, as is determining the appropriate surgical approach and whether any pre-/post-surgical radiotherapy or other adjuvant/neoadjuvant therapies are needed around the time of surgery [11]. Notably, the MARS set of clinical trials tried to determine whether surgery is beneficial in mesothelioma. In the first MARS trial, the authors found that extrapleural pneumonectomy (EPP) did not benefit the patients and may have been harmful due to its high morbidity [12]. Moreover, in MARS 2, a different surgical approach of extended pleurectomy decortication (EPD) in combination with chemotherapy led to worse survival rates, increased adverse events, and reduced quality of life, discouraging clinicians from performing surgery in mesothelioma patients [13]. Still, more clinical data are needed to conclude that surgery is not a viable option for this condition. The addition of hyperthermic intraoperative chemotherapy (HITHOC) to the surgical approach has been observed in various prospective and retrospective studies, but no large clinical trials have been conducted for this strategy [14]. HITHOC combines several therapeutic options, including surgery, intraoperative and topical administration of chemotherapeutic drugs, and simultaneous warming of the thoracic cavity, to treat cancer cells that spread to the pleural spaces. The adverse events reported for HITHOC include local pain from the procedure; nausea and vomiting; anemia and thrombocytopenia; and even hemodynamic and respiratory instability, in rare cases [14]. However, the variability in the actual indications and technical applications of HITHOC currently limits its clinical usage. Palliative systemic therapy via chemotherapy is the treatment of choice for most patients, with the combination of cisplatin and pemetrexed being preferred. Radiotherapy can also be used in palliative settings to manage symptoms that are not manageable with drugs [11]. Until relatively recently, clinicians utilized the options mentioned above to treat mesothelioma patients, which contributed to their poor prognosis. As discussed below, the evolution of our understanding of how malignancies such as mesothelioma function has enabled various breakthroughs and innovations related to therapeutic options. In the next section, we discuss the novel therapies that are approved, being examined, or being considered for mesothelioma treatment. A relatively recent development in the usage of immunotherapy has changed the landscape of mesothelioma treatment. Significant improvements could be seen after its administration in several large clinical trials [15,16]. Beyond immunotherapy, many other novel therapeutic strategies are being developed to combat mesothelioma. Below, we discuss in detail the current evidence for these strategies. It must be noted, however, that the only novel treatment options currently approved for clinical use are nivolumab/ipilimumab combination immunotherapy, nivolumab monotherapy (in Japan), and tumor-treating fields (TTFields). Table 1 summarizes the novel therapeutic modalities for mesothelioma that are discussed below.

## 3. Novel Therapeutic Modalities for Mesothelioma

### 3.1. Immunotherapy

Immunotherapies targeting immune checkpoint molecules such as PD-1 (programmed cell death 1), PD-L1 (programmed cell death 1-ligand 1), and CTLA-4 (cytotoxic T-lymphocyte-associated antigen 4) may be effective treatments for mesothelioma [17]. Targeting these molecules, which can control the survival of cancer cells, using immune checkpoint inhibitors (ICIs) has recently gained traction in various types of cancer, especially solid tumors [18]. In the case of mesothelioma, clinical trials investigating ICI usage have been ongoing for the last 15+ years with promising results in various settings.

Combination therapies involving a PD-1 inhibitor and a CTLA-4 inhibitor or kinase-targeting therapies are gaining traction as viable strategies to treat mesothelioma, so much so that one of the combinations was approved for clinical use as a first-line therapy. The combination of the PD-1 inhibitor nivolumab and the CTLA-4 inhibitor ipilimumab has been the most extensively studied so far. First, in the INITIATE trial, 240 mg of nivolumab biweekly was combined with 1 mg/kg of ipilimumab every 6 weeks in 34 patients who relapsed after platinum-based chemotherapy [19]. Encouragingly, 10 patients had partial responses, and 13 were stable, bringing the DCR to 68%, with the most common adverse events being infusion-related reactions, skin disorders, and fatigue. Responses to this therapy were seen more in those with high tumor PD-L1 expression. Similarly, the MAPS2 phase 2 trial also observed the effect of nivolumab when used in combination with ipilimumab or as a monotherapy. Here, the authors showed that the nivolumab/ipilimumab combination achieved a higher ORR (28% vs. 19% for the monotherapy), median OS (15.9 months vs. 11.9 months), and median PFS (5.6 months vs. 4.0 months) [20]. Adverse events included asthenia and asymptomatic increases in liver enzymes and lipase. Expanding the study of nivolumab/ipilimumab combination therapy in mesothelioma to a bigger setting, the CheckMate-743 phase III trial enrolled 713 patients with previously untreated, unresectable mesothelioma from 21 countries and compared nivolumab/ipilimumab combination therapy to chemotherapy. The ICI combination therapy was found to be superior to chemotherapy in improving the median OS (18.1 months vs. 14.1 months) and the duration of the response (11.0 months vs. 6.7 months), with similar median PFS (6.8 months vs. 7.2 months) and ORR (40% vs. 43%) results [21]. The occurrence rates of grade 3–4 adverse events, which included pneumonitis and encephalitis, were also similar between the two arms. Again, those with high PD-L1 expression were observed to benefit more from this immunotherapy regimen. The 3- and 4-year follow-up studies of CheckMate-743 showed the consistent superiority of the nivolumab/ipilimumab combination therapy compared to chemotherapy in terms of prolonging survival, with a 3-year OS rate of 23% vs. 15% for the monotherapy and a 4-year OS rate of 16.8% vs. 10.7% for the monotherapy. Notably, the 3-year PFS rate was also markedly higher (14% vs. 1%) [22,23]. As such, the nivolumab/ipilimumab combination therapy has been approved by the FDA (the U.S. Food and Drug Administration) and the EMA (the European Medicines Agency) as a first-line therapy for unresectable malignant mesothelioma.

As a monotherapy, nivolumab, an ICI targeting the PD-1/PD-L1 pathway, has also been used clinically in certain countries. The NivoMes trial first showed how nivolumab monotherapy (3 mg/kg biweekly for up to 12 months) could be used effectively in mesothelioma patients [24]. Here, 12 weeks of nivolumab treatment successfully helped 47% of the treated patients achieve an appropriate disease control rate as determined by the trial. Notably, out of 34 patients, 8 achieved partial responses, and another 8 had stable disease conditions [24]. Fatigue and pruritus were the most common adverse events, while grade 3–4 adverse events included pneumonitis and gastrointestinal disorders. The phase 2 single-arm MERIT trial from Japanese investigators was another relative success story for nivolumab monotherapy in advanced, unresectable mesothelioma. In 34 patients treated with 240 mg of nivolumab biweekly, the median PFS was 6.1 months with a median OS of 17.3 months and 47% experiencing adverse events of grade 3 or higher such as interstitial pneumonia or pneumonitis. Notably, those with high PD-L1 expression had higher ORR, median PFS, and median OS values [25]. A 3-year follow-up study of MERIT confirmed increases in the 2-year and 3-year OS rates (35.3% and 23.5%, respectively) [26]. The CONFIRM phase 3 trial also showed similar efficacy for nivolumab, this time compared with a placebo, in 332 previously treated patients. Here, the nivolumab treatment significantly improved the median PFS and OS values, with similar rates of serious adverse events compared to the placebo arm [27]. Dyspnea, pneumonia, and diarrhea were the most common serious adverse events in the nivolumab arm. While a study of 107 patients in the Netherlands observed lower median PFS and OS values, it was also noted that survival rates were higher among those with high PD-L1 expression [28]. Buoyed by the efficacy shown in these trials, nivolumab was approved as a monotherapy in Japan, and further trials assessing its efficacy in different clinical settings are currently planned or ongoing [29,30].

Meanwhile, pembrolizumab was the first PD-1 inhibitor tested for mesothelioma in the KEYNOTE series of trials for ICIs. In the mesothelioma branch of KEYNOTE-028, 35 advanced mesothelioma patients that did not respond to the standard therapy received biweekly doses of 10 mg/kg of pembrolizumab for up to two years. In total, 20% of the patients achieved an objective response, while 52% of all patients achieved stable disease conditions. More encouragingly, no major treatment-related adverse events were observed during this study [31]. Expanding on this study, the phase 2 KEYNOTE-158 trial enrolled 118 patients with previously treated mesothelioma, who received 200 mg of pembrolizumab every 3 weeks for up to 35 cycles. The median overall survival (OS) was 10 months, and the median progression-free survival (PFS) was 2.1 months. In total, 10 out of 118 patients (8.47%) showed an objective response, with a median response duration of 14.3 months. The objective responses were observed in six patients with PD-L1-positive tumors and four with PD-L1-negative tumors, with notable adverse events including rhabdomyolysis, hypothyroidism, erythema, and iridocyclitis [32]. The PROMISE-MESO trial compared 200 mg of pembrolizumab every three weeks with chemotherapy in 144 platinum-treated mesothelioma patients and found that only the objective response rate (ORR) was higher in the pembrolizumab arm (22% vs. 6% for chemotherapy), while no significant improvements were found in the median PFS and OS [33]. Nausea, oral mucositis, and constipation were common adverse events in the treatment arm. More recently, pembrolizumab was combined with chemotherapy (cisplatin/pemetrexed) in an open-label, phase 3 multinational trial of 440 patients that were randomly assigned to pembrolizumab addition or chemotherapy alone. The authors found increases in the median OS (17.3 vs. 16.1 months) and the 3-year OS rate (25% vs. 17%), although an increase in grade 3 or 4 adverse events was also observed in the pembrolizumab arm [34]. Currently, the ongoing CHIMERA phase 2 trial is investigating pembrolizumab in combination with platinum-based chemotherapy as a neoadjuvant therapy (NCT06155279). Overall, pembrolizumab seems to have the potential to become a staple of mesothelioma treatment in the near future.

Many different kinds of PD-1 inhibitors are also being investigated as treatments for mesothelioma. In the JAVELIN phase 1 study, biweekly treatment with 10 mg/kg of the PD-1 inhibitor avelumab was found to give responses in 5 out of 53 patients (9% ORR), again with better responses in the PD-L1-positive patients [35]. When combined with stereotactic body radiation therapy (SBRT), avelumab also showed tolerability and safety in a phase 1 trial in the USA, where only one of thirteen patients administered avelumab + SBRT developed grade 3+ adverse events, which included pneumonitis, colitis, and hyperthyroidism. Durvalumab is another PD-1 inhibitor, and its efficacy when combined with chemotherapy has been investigated in several trials. The phase 2 DREAM trial analyzed the effect of adding 1125 mg of durvalumab to cisplatin and pemetrexed every three weeks, with only the durvalumab treatment continued as maintenance. In 54 patients that were followed for a median of 28.2 months, the 6-month PFS rate was 57%, while the median PFS was 6.9 months, with an ORR of 48% and adverse events related to treatment occurring in five patients [36]. Another phase II trial, PrE0505, evaluated 55 malignant mesothelioma patients treated with first-line durvalumab + platinum–pemetrexed chemotherapy and then maintenance durvalumab as a single arm. This combination therapy achieved a median OS of 20.4 months compared to 12.1 months in the historical chemotherapy-only control, with an estimated 12-month OS rate of 70.4%. The median PFS was 6.7 months, and the ORR was 56.4%. Additional analysis showed that a higher immunogenic mutation burden coupled with a more diverse T-cell repertoire was linked to favorable clinical outcomes and that patients with germline alterations in cancer-related genes were more responsive to this combination treatment and more likely to be longer-term survivors [37]. This study also mentioned that most of the adverse events were lower-grade, including fatigue, nausea, and anemia. Higher-grade adverse events included hyponatremia, leucopenia, and thrombocytopenia. In a different setting involving DIADEM-treated patients that progressed after platinum/pemetrexed chemotherapy with 1500 mg of durvalumab every 4 weeks for up to 12 months, it was observed that from 69 patients, only 17 were alive or free of progression after 16 weeks. In addition, only six patients could complete the 12-month regimen. This indicated that as a standalone therapy, it could not achieve the same efficacy as when combined with chemotherapy. Phase 3 studies for DREAM and PrE0505 (called DREAM3R and PrE0506, respectively) are ongoing. Other ongoing studies for PD-1 and PD-L1 inhibitors, such as the PD-L1 inhibitor atezolizumab, include the AtezoMeso and BEAT-meso trials.

The CTLA-4 inhibitor tremelimumab was one of the first ICIs trialed for mesothelioma in the MESOT-TREM-2008 study [38]. Here, the authors reported partial positive responses to tremelimumab administered every 90 days in 2 out of 29 unresectable malignant mesotheliomas after first-line platinum therapy that lasted up to 18 months [38]. The MESOT-TREM-2012 study tried a more intensive regimen (10 mg/kg every 4 weeks for six doses and then every 12 weeks) and found that 52% of the patients achieved disease control, with a median OS of 11.3 months and a median PFS of 6.7 months [39]. Following up on those studies, the double-blind DETERMINE trial enrolled a higher number of patients (571) with previously treated advanced mesothelioma that were randomized to receive either tremelimumab or a placebo [40]. Unfortunately, this trial did not reach its original endpoint due to the deaths of >80% of the patients enrolled in both arms. At the time of the data cutoff, the median OS values were not significantly different between the tremelimumab (7.7 months) and placebo (7.3) arms [40]. The overall risk reduction was calculated to be only 4.5%, but an additional analysis showed that patients showing the sarcomatoid subtype might benefit more from tremelimumab [40]. Adverse events reported with tremelimumab include cutaneous rashes, pruritus, colitis, and diarrhea.

While tremelimumab has not shown high efficacy as a standalone therapy, more recently, tremelimumab was combined with other ICIs with a more promising result. In the single-arm NIBIT-MESO-1 trial, tremelimumab was combined with durvalumab for the first four weeks in 40 malignant mesothelioma patients, who then continued with maintenance durvalumab [41]. The median OS was 16.5 months, with 20% of the patients surviving at 36 months. The authors also re-treated 17 patients meeting the criteria and found an additional median OS of 12.5 months. The authors also noted that a higher tumor mutation burden (TMB) was associated with longer survival [41]. Extrapolating from this potential efficacy, a phase I study observing the safety of tremelimumab as a monotherapy or in combination with durvalimumab was commenced in Japan with positive results [42]. Further studies are warranted to confirm the effectiveness of tremelimumab in treating mesothelioma in a combined setting. Table 2 summarizes the current trials of ICIs for treating mesothelioma.

### 3.2. Molecular Targeted Therapy

Several molecules have been found to be important in the progression of mesothelioma, and advancements in drug development allow for some of these molecules to be targeted as a therapeutic option. One such molecule is vascular endothelial growth factor (VEGF) [43]. The role of VEGF in malignancy is not limited to mesothelioma and extends to many other solid tumors. This is due to its chief function of promoting angiogenesis, which is highly conducive to the progression of cancer cells. As such, it is understandable that the use of VEGF inhibitors is thought to be beneficial in mesothelioma. The phase 3 MAPS trial is one study that successfully showed the efficacy of VEGF inhibition by the drug bevacizumab in combination with cisplatin and pemetrexed compared to treatment without bevacizumab. As a result, the median OS was significantly improved in the bevacizumab arm (18.8 months vs. 16.1 months with chemotherapy alone) [44]. Notably, hypertension and thrombosis were more frequent in the bevacizumab arm. Bevacizumab also showed durable efficacy in a phase 2 trial by a team from the University of Texas, U.S.A., when combined with the PD-L1 inhibitor atezolizumab. Here, the investigators found that among 20 treated patients, 40% showed an objective response, with a 1-year PFS rate of 61% and a 1-year OS rate of 85% [45]. Hypertension (40%) and anemia (10%) were the most common adverse events. Beyond bevacizumab, the RAMES trial investigated ramucirumab, a monoclonal antibody for VEGF receptor (VEGFR) 2. In total, 161 inoperable malignant mesothelioma patients were randomized to receive either ramucirumab or a placebo in combination with gemcitabine. The ramucirumab arm showed improved median OS (13.8 months vs. 7.5 months for the placebo) and median PFS (6.4 months vs. 3.3 months) results [46]. Again, hypertension was an adverse event found in the treatment arm in addition to neutropenia. Another agent that targets VEGF receptors is the tyrosine kinase inhibitor nintedanib, which can also target other receptors such as fibroblast growth factor receptors (FGFR1-3) and platelet-derived growth factor receptors (PDGFRα/β). The LUME-Meso trial was a phase II/III trial. In the phase II trial, 87 patients were randomized to receive nintedanib or a placebo in addition to cisplatin–pemetrexed [47]. The median PFS was improved in the nintedanib arm (7.8 months vs. 5.3 months), which helped this study to continue to phase III. In the phase III trial, however, similar improvements in the median PFS due to nintedanib were not seen (6.8 months vs. 7.0 months) in a larger sample of 458 patients [48]. Lastly, cediranib, another tyrosine kinase inhibitor that affects VEGFRs, was assessed in a phase II trial where patients previously treated with chemotherapy were randomized to receive either cediranib or a placebo. While the cediranib treatment improved the median PFS, its safety profile was worse compared to the placebo, with more grade 3–4 adverse events, such as neutropenia [49]. Similarly, in the SWOG S0905 trial, where cediranib was added to chemotherapy, a worse safety profile in the form of an increase in grade 3–4 adverse events (diarrhea, dehydration, hypertension, and weight loss) was observed in the cediranib arm compared to chemotherapy only, even with an increase in the median PFS (7.2 months vs. 5.6 months) [50].

Mesothelin is another molecule that has recently been frequently targeted in various forms of solid, epithelial cancers, including mesothelioma. Mesothelin is a glycoprotein, and its role as a biomarker in mesothelioma has been reviewed elsewhere. Its function in cancer progression is thought to involve the promotion of cancer cell proliferation, growth, and adhesion [51,52]. Amatuximab is a monoclonal antibody that neutralizes mesothelin, and recent studies have shown its efficacy in treating malignant mesothelioma. A phase II trial involving 89 patients showed that when administered in combination with platinum-based chemotherapy, amatuximab improved the median OS of mesothelioma patients (14.8 months) and had an ORR of 90% [53]. Importantly, eleven patients exhibited amatuximab-related hypersensitivity reactions. Similar to amatuximab, anetumab ravtansine is another monoclonal antibody against mesothelin. It is conjugated to DM4, which is internalized by mesothelin-expressing cancer cells. The phase II ARCS-M trial studied the use of this drug in unresectable mesothelioma overexpressing mesothelin. Unfortunately, no significant differences in the median PFS or the median OS were found in the anetumab ravtansine arm compared to the vinorelbine arm. In addition, ten treatment-emergent deaths occurred in the anetumab ravtansine arm, caused by pneumonia, dyspnea, sepsis, and other conditions [54]. A recent trial that combined anetumab ravtansine with pembrolizumab also showed negative data, where no changes in the ORR were observed between the combination arm and the pembrolizumab-only arm [55]. Lastly, SS1P targets mesothelin in a different way, as it is an anti-mesothelin immunotoxin. While in phase 1 studies, SS1P was well tolerated by inoperable, untreated mesothelioma patients, no further studies have been conducted so far for this specific agent [56].

Beyond the two major molecules, several others are also being targeted for mesothelioma. PARP is a DNA damage sensor with roles in cell repair and apoptosis, and expression of PARP increases after asbestos exposure and in mesothelioma cancer cells, making it an attractive therapeutic target [57]. Two different PARP inhibitors have been tested clinically. First, olaparib was recently analyzed in a phase 2 trial, but no significant improvements were seen in the median PFS (3.6 months) or the median OS (8.7 months), and common adverse events included nausea, renal toxicity, and fatigue [58]. Another PARP inhibitor, niraparib, was also investigated in various tumors harboring mBAP1 mutations, including mesothelioma. In total, 6% of patients achieved partial responses, while 8% had stable disease conditions, and this treatment had a good safety profile [59]. On the other hand, EZH2 is another DNA damage-related molecule, and inhibiting it with tazemetostat was tested in a phase 2 trial. A disease control rate of 54% was observed, but only 2 out of 74 patients had partial responses to the therapy [60]. It is clear that targeted molecular therapy, while promising, still has a way to go to achieve clinical usability.

### 3.3. Cell Therapy

Recent breakthroughs in biomedical engineering have made cell-based therapies possible in various clinical conditions. Mesothelioma is one such condition that could benefit from this technology, and various cellular therapies have been or are currently being investigated to treat it. One example is chimeric antigen receptor (CAR)-T cell therapy. CAR-T cell therapy is a novel strategy where autologous or donor T cells are genetically modified to express a chimeric tumor cell antigen-specific receptor on their cell surfaces, aiming to elicit an anti-tumor immunologic response from the body [61]. Several trials have preliminarily examined how CAR-T-based therapy could treat mesothelioma. First, a phase 1 trial examining the use of anti-mesothelin M5 CAR cells in solid tumor patients, including mesothelioma patients, found that, following infusion, 8 out of 14 patients showed stable disease conditions with good treatment tolerability, although no clinical changes were observed [62]. Another phase 1 trial that combined regional anti-mesothelin CAR-T cells with pembrolizumab found that the median OS was 23.9 months, with eight patients in stable condition after 6 months. A phase I trial of an anti-CD28-costimulated CAR-T-targeting fibroblast-activating protein (FAP), a cell-surface antigen highly expressed in epithelial cancers, enrolled three patients with mesothelioma and at least two prior lines of treatment and delivered the therapy intrapleurally. It was found to be safe, with two of the three patients alive 18 months after the treatment [63]. Lastly, a phase 1 trial analyzed the use of the MSLN-targeted CAR-T therapy gavocabtagene autoleucel (gavo-cel), which fuses an anti-mesothelin antibody to a glycine/serine spacer and a human CD3ε subunit. It found similar acceptable safety profiles for the CAR-T cells and a high level of disease control [64]. Further studies, such as the phase I/II EVEREST-2 study and a phase II trial for gavo-cel, are currently ongoing [65].

**Figure 1:**
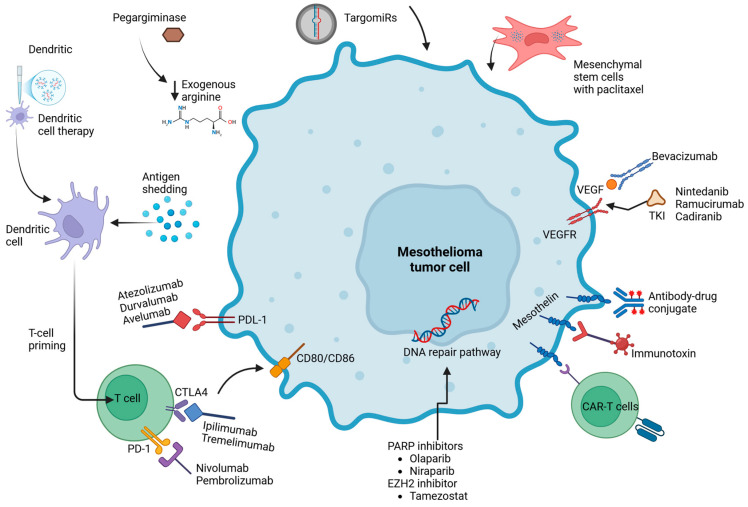
A summary of the novel therapeutic targets for mesothelioma. Created using Biorender.com.

Dendritic cell (DC) therapy is another interesting cell-based approach to treating mesothelioma [66]. It is carried out by harvesting and differentiating an autologous or donor DC precursor ex vivo, exposing the differentiated DC to cancer antigens, and re-injecting those cells to stimulate anti-tumor immune responses [67]. Several phase 1 trials have been conducted using this therapeutic method in mesothelioma patients. One showed a median OS of 19 months when DC therapy was used as a consolidative therapy after chemotherapy [68]. Another phase 1 trial, the MesocancerVA trial, found a median PFS of 8.8 months in nine patients treated with autologous DCs with good tolerability [69]. The phase II/III DENIM trial then recruited a larger number of patients treated using a similar DC therapy with up to five infusions, but the median OS in the DC infusion arm was 16.8 months compared to 18.3 months in the best supportive care arm, putting the efficacy of this DC treatment in question [70]. Adverse events included chest pain, dyspnea, anemia, and pneumonia. The authors concluded that ICIs are still the standard of care and that future studies should aim to investigate DCs in combination with ICIs, which is currently ongoing in the MESOVAX trial [71]. Figure 1 summarizes the novel therapeutic targets for mesothelioma currently being studied clinically.

### 3.4. Other Novel Therapies for Mesothelioma

Beyond the more established innovations in mesothelioma therapies, other groups have also developed various ways to combat the poor prognosis of mesothelioma by utilizing advances in biomedical research to directly halt the progression of cancer cells. Tumor-treating fields (TTFields) are one such advance. They utilize low-intensity, alternating 150 kHz electric fields to prevent cell mitosis and induce cytotoxicity. The STELLAR single-arm phase 2 trial successfully showed that when paired with chemotherapy, TTFields can improve the prognosis of mesothelioma patients [72]. The median OS of 80 patients after treatment with TTFields and chemotherapy was 18.2 months, with skin reactions being the most common adverse events related to TTFields (occurring in 71% of the patients) [72]. The results of the STELLAR trial led the FDA to approve TTFields therapy for use in mesothelioma. It must be noted, however, that high financial costs and compromised quality of life due to the continuous nature of TTFields therapy impact the utilization of this therapeutic modality clinically.

One other solution being tested is to deliver specific agents to genetically modify cancer cells to inhibit them or even induce their destruction. For example, an RNA delivery strategy that has been widely used in various experimental procedures can now be utilized to silence unwanted genes in cancer cells. One such example is DFP-10825. DFP-10825 is a thymidylate synthase (TS)-targeting short hairpin RNA (shRNA) that definitively silences TS, which is important in cancer cells for growth and proliferation. However, DFP-10825 is still in the preclinical phase [73]. Another strategy is to replace lost micro-RNAs (miRNAs). This is the strategy employed by TargomiRs, which are minicells (EnGeneIC Dream Vectors) loaded with miR-16-based mimic miRNA to counteract the loss of miR-16 in mesothelioma cancer cells. A phase 1 trial for TargomiRs showed an acceptable safety profile with 1 partial responder out of 22, while 15 achieved stable disease conditions [74]. Larger clinical trials are expected in the future for these gene-modifying therapies.

Mesothelioma has also been associated with a loss of arginosuccinate synthetase 1, an enzyme in the urea cycle that can work as a tumor suppressor. Without this enzyme, cancer cells become reliant on arginine to survive and grow. To this end, depleting arginase using pegargiminase has been studied, and recently, the ATOMIC-Meso randomized phase III trial showed that it could be effective in mesothelioma. In this study, 249 chemotherapy-naïve mesothelioma patients were administered pegargiminase in combination with platinum and pemetrexed chemotherapy or chemotherapy alone. The addition of pegargiminase improved the median OS to 9.3 months compared to 7.7 months in the chemotherapy-only arm [75]. The adverse events during the pegargiminase treatments included nausea, fatigue, and constipation, while reactions of grade 3 or higher included neutropenia, anaphylactic hypersensitivity, and skin reactions. This may represent a new treatment strategy for mesothelioma patients.

Lastly, the use of mesenchymal stem/stromal cells (MSCs) is a recent but rapidly developing tool to either induce repair in injured tissue or, in the case of cancers such as mesothelioma, deliver specific agents needed to destroy tumor cells. To this end, the phase I PACLIMES trial plans to evaluate the effects of injections of MSCs loaded with paclitaxel into the pleural spaces of mesothelioma patients as a neoadjuvant treatment [76].

## 4. Conclusions

Novel strategies for treating mesothelioma patients, especially those with advanced, malignant, and inoperable cases, are starting to emerge and to be proven useful in clinical studies. This is especially true for immunotherapies, some of which have been used and approved clinically. Other modalities for treating mesothelioma are also being developed with the hope that more diverse options for treating mesothelioma can lead to a better prognosis for patients in the near future.

## Figures and Tables

**Table 1:** A summary of the novel therapeutic modalities for mesothelioma.

Modality	Mechanism of Work	Current Status
Immune checkpoint inhibitors	Inhibit PD-1/PD-L1 or CTLA-4, which can be used by cancer cells to escape immune cell-related destruction	Accepted for clinical use (nivolumab/ipilimumab, nivolumab); experimental phase (others)
VEGF inhibitor	Inhibits VEGF, which promotes angiogenesis and the growth of tumors	Experimental phase
Mesothelin inhibitor	Inhibits mesothelin-mediated promotion of cancer cell proliferation, growth, and adhesion	Experimental phase
PARP inhibitor	Inhibits PARP-mediated DNA damage response	Experimental phase
EZH2 inhibitor	Inhibits EZH2-mediated DNA damage response	Experimental phase
CAR-T cells	Modified T cells expressing chimeric tumor cell antigen-specific receptors on their cell surfaces to elicit an anti-tumor response	Experimental phase
Dendritic cell therapy	Harvesting and differentiating a DC precursor ex vivo, exposing it to cancer antigens, and re-injecting those cells to stimulate anti-tumor responses	Experimental phase
TargomiR	Minicells (EnGeneIC Dream Vectors) loaded with miR-16-based mimic miRNA	Experimental phase
Pegargiminase	Arginase depletion for cancer cells	Experimental phase
Mesenchymal stromal cell-assisted paclitaxel delivery	Delivers chemotherapy agents directly to tumor cells	Experimental phase
Tumor-treating fields	Exposure to low-intensity, alternating 150 kHz electric fields to prevent cell mitosis and induce cytotoxicity	Accepted for clinical use

**Table 2:** A summary of the major clinical trials of ICIs for treating mesothelioma.

Drug	Trial	Control	Phase	Main Results
Pembrolizumab	KEYNOTE-158	None	2	Median OS: 10 months, median PFS: 2.1 months, ORR: 8.5%
Pembrolizumab + Chemotherapy	PROMISE-MESO	Chemotherapy	3	ORR: 22% (vs. 6%), median PFS: 2.5 months (vs. 3.4 months), median OS: 10.7 months (vs. 12.4 months)
Nivolumab	NivoMes	None	2	DCR: 47%
Nivolumab	MERIT	None	2	Median PFS: 6.1 months, median OS: 17.3 months
Nivolumab	CONFIRM	Placebo	3	Median PFS: 3.0 months (vs. 1.8 months), median OS: 10.2 months (vs. 6.9 months)
Nivolumab + Ipilimumab	INITIATE	None	2	DCR: 68%
Nivolumab + Ipilimumab	MAPS2	Nivolumab	2	ORR: 28% (vs. 19%), median PFS: 5.6 months (vs. 4.0 months), median OS: 15.9 months (vs. 11.9 months)
Nivolumab + Ipilimumab	CheckMate-743	Chemotherapy	3	Median OS: 18.1 months (vs. 14.1 months), median PFS: 6.8 months (vs. 7.0 months), duration of response: 11 months (vs. 6.7 months)
Avelumab	JAVELIN	None	1	ORR: 9%
Durvalumab + Chemotherapy	DREAM	None	2	6-month PFS rate: 57%, median PFS: 6.9 months, ORR: 48%
Durvalumab + Chemotherapy	PrE0505	Historical chemotherapy	2	Median OS: 20.4 months (vs. 12.1 months), median PFS: 6.7 months, ORR: 56.4%
Durvalumab	DIADEM	None	2	16-week OS rate: 24.6%
Tremelimumab	MESOT-TREM-2008	None	2	DCR: 31%, median PFS: 6.2 months, median OS: 10.7 months
Tremelimumab	MESOT-TREM-2012	None	2	DCR: 52%, median OS: 11.3 months, median PFS: 6.7 months
Tremelimumab	DETERMINE	Placebo	2b	Median OS: 7.7 months (vs. 7.3 months)
Tremelimumab + Durvalumab	NIBIT-MESO-1	None	2	Median OS: 16.5 months, ORR: 28%, median PFS: 5.7 months

## Abbreviations

ICI	immune checkpoint inhibitor
CAR-T	chimeric antigen receptor-T
TNF-α	tumor necrosis factor α
NF-κB	nuclear factor κB
PD-1	programmed cell death 1
PD-L1	programmed cell death 1-ligand 1
CTLA-4	cytotoxic T-lymphocyte-associated antigen 4
OS	overall survival
PFS	progression-free survival
ORR	objective response rate
SBRT	stereotactic body radiation therapy
FDA	U.S. Food and Drug Administration
EMA	European Medicines Agency
TMB	tumor mutation burden
VEGF	vascular endothelial growth factor
VEGFR	vascular endothelial growth factor receptor
FGFR	fibroblast growth factor receptor
PDGFR	platelet-derived growth factor receptor
DC	dendritic cell
shRNA	short hairpin ribonucleic acid
miRNA	micro ribonucleic acid
MSC	mesenchymal stromal cells
TTFields	tumor-treating fields
